# Generation of *NSE-MerCreMer* Transgenic Mice with Tamoxifen Inducible Cre Activity in Neurons

**DOI:** 10.1371/journal.pone.0035799

**Published:** 2012-05-07

**Authors:** Mandy Ka Man Kam, King Yiu Lee, Paul Kwong Hang Tam, Vincent Chi Hang Lui

**Affiliations:** 1 Department of Surgery, LKS Faculty of Medicine, The University of Hong Kong, Hong Kong SAR, China; 2 Centre for Reproduction, Development & Growth, LKS Faculty of Medicine, The University of Hong Kong, Hong Kong SAR, China; University of South Florida, United States of America

## Abstract

To establish a genetic tool for conditional deletion or expression of gene in neurons in a temporally controlled manner, we generated a transgenic mouse (*NSE*-*MerCreMer*), which expressed a tamoxifen inducible type of Cre recombinase specifically in neurons. The tamoxifen inducible Cre recombinase (MerCreMer) is a fusion protein containing Cre recombinase with two modified estrogen receptor ligand binding domains at both ends, and is driven by the neural-specific rat neural specific enolase (*NSE*) promoter. A total of two transgenic lines were established, and expression of MerCreMer in neurons of the central and enteric nervous systems was confirmed. Transcript of *MerCreMer* was detected in several non-neural tissues such as heart, liver, and kidney in these lines. In the background of the Cre reporter mouse strain *Rosa26R*, Cre recombinase activity was inducible in neurons of adult *NSE-MerCreMer* mice treated with tamoxifen by intragastric gavage, but not in those fed with corn oil only. We conclude that *NSE-MerCreMer* lines will be useful for studying gene functions in neurons for the conditions that Cre-mediated recombination resulting in embryonic lethality, which precludes investigation of gene functions in neurons through later stages of development and in adult.

## Introduction

Conventional gene knockout in mice can result in early lethality, which prevents study of gene functions through later stages of development and in adult. To circumvent early lethality, the use of *Cre/loxP* system to generate conditional gene deletion in a spatial and temporal manner has become the most popular method. Cre recombinase is a Type I topoisomerase from bacteriophage P1 that catalyzes the site-specific recombination of DNA between *loxP* elements in both bacteriophage and mammalian cells [1], [2], [3]. The *loxP* recognition element is a 34 base pair (bp) DNA sequence comprised of two 13 bp inverted repeats flanking an 8 bp spacer region which confers directionality [4]. Cre recombination could result in excision, inversion or translocation of the floxed (flanked with two *loxP* sites) genomic regions depending on the locations and orientations of the *loxP* elements [3], [5]. By utilization of a specific promoter driving a spatially restricted Cre expression, conditional deletion of a gene or activation of transgene expression can be achieved in specific tissue in mice, thus offering the opportunity to study gene function with spatial control [3], [6]. A further refinement of the *Cre*/*loxP* technology is the development of inducible Cre transgene which permits temporal control of gene recombination, allowing investigation of gene functions in a particular developmental stage of the entire life-span of mice.

The inducible Cre recombinase is consisted of mutated ligand-binding domain (LBD) of the mouse estrogen or progesterone receptor and Cre recombinase [3], [7], [8]. The mutated LBD fails to bind to estrogen or progesterone, but retains its ability in binding to synthetic ligands such as tamoxifen, 4-OHT (4-hydroxy-tamoxifen) and RU486 [3], [7], [9]. When ligand is absent, LBD-Cre is bound by HSP90 and retained in the cytoplasm. Upon ligand binding, LBD-Cre translocates into the nucleus and mediates genomic recombination [3], [7], [9], [10]. Therefore, Cre-mediated recombination is induced by the administration of synthetic ligand, allowing a temporal control of the recombination event [3], [11]. The tamoxifen inducible Cre recombinase protein (MerCreMer) is composed of (i) Cre recombinase (Cre) and (ii) two tamoxifen-binding domains (Mer) of mutated mouse estrogen receptor α (ERα), one at each end of the Cre recombinase, ensuring efficient binding of MerCreMer to tamoxifen and 4-OHT, but at the same time retaining maximal Cre activity [9].

Neural specific enolase (NSE) is a glycolytic enzyme enolase abundantly but specifically expressed in terminally differentiated neurons and neuroendocrine cells [12]. Transcript of mouse *NSE* was detectable from E12 onwards [13], and its expression was correlated with synaptogenesis [12], [14]. The 1.8 kb rat *NSE* promoter DNA fragment has been shown to drive expression of target genes in brain neurons of the transgenic mice [13], [15], [16], [17], [18], [19]. A *NSE-Cre* mouse line has been previously generated which exhibited spatially restricted Cre activity in neurons of the central nervous system [18]. However, such mouse line did not allow temporal control of Cre recombination.

In this study, we generated transgenic mouse line (*NSE-MerCreMer*) that expressed tamoxifen inducible Cre activity in neurons. We cloned the rat *NSE* promoter 5′ to the cDNA encoding the *MerCreMer* and generated transgenic mouse lines that expressed tamoxifen inducible Cre activity in neurons. RT-PCR, Western blot analysis, immuno-histochemistry for *MerCreMer* were performed to investigate the temporal and spatial expression patterns of *MerCreMer* in different transgenic lines. Furthermore, we crossed our mouse lines to reporter mice *Rosa26R* (*R26R*) [20]. Immuno-staining for *β*-galactosidase in *NSE-MerCreMer*/*R26R* mice showed that Cre activity in neurons of the central and enteric nervous systems was induced by tamoxifen.

## Results and Discussion

### Establishment of NSE-MerCreMer Transgenic Mice

**Figure 1:**
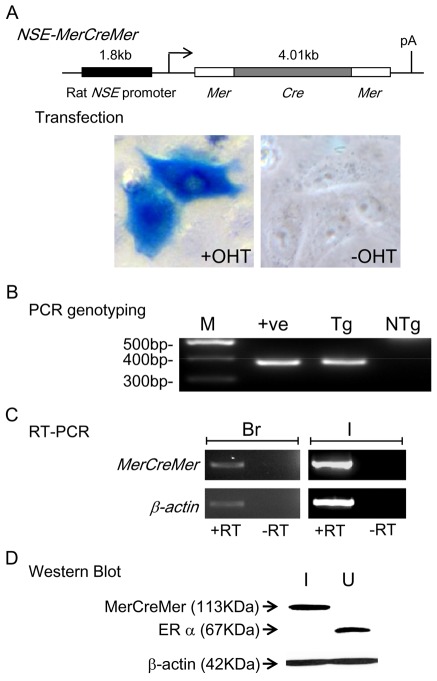
Generation of *NSE-MerCreMer* transgenic lines. A, Transgenic construct *NSE-MerCreMer* consists of a 1.8 kb rat *NSE* gene promoter and the cDNA encoding the tamoxifen inducible Cre recombinase (MerCreMer). X-gal staining of *NSE-MerCreMer* and *pCAG-CAT-LacZ* co-transfected HeLa cells with (+OHT) or without (−OHT) the addition of synthetic ligand 4-OHT. B, PCR amplification of *NSE-MerCreMer* transgene using *CreR1* and *CreF1* primer pair generated a 374 bp DNA fragment from genomic DNA of *NSE-MerCreMer* transgenic mice (Tg) but not from non-transgenic mice (NTg). ‘+ve’ denotes PCR amplification using the transgenic construct as template DNA. C, RT-PCR analysis showed expression of the transgene (*MerCreMer*) in the brain (Br, +RT) and intestine (I, +RT) of transgenic mouse. RT-PCR for mouse *β*-actin (*β*-*actin*) was included to check the integrity of the RNA. Reverse transcriptase was omitted in the first strand cDNA synthesis which served as a negative control (−RT) to ascertain the PCR product was not amplified from genomic DNA. D, Western blot analysis using anti-body against ligand binding domain of estrogen receptor α (ERα) detected a protein band of MerCreMer (113 kDa) in the intestine (I), and a protein band of ERα (67 kDa) in the uterus (U) of transgenic mouse. Abbreviation: M, DNA size marker.

Transgenic construct *NSE-MerCreMer* was constructed by linking the rat *NSE* promoter 5′ upstream of the cDNA encoding the tamoxifen inducible Cre recombinase (MerCreMer), which is composed of Cre recombinase and mutated ligand-binding domain (LBD) of the mouse estrogen receptor α (ERα) (Mer) on both ends (Figure 1). MerCreMer will only translocate into the nucleus and mediate genomic recombination in the presence of synthetic ligands such as tamoxifen and 4-OHT [3], [7], [9], [10]. To ascertain the tamoxifen induction of Cre activity of *NSE-MerCreMer*, the transgenic construct was co-transfected together with the *pCAG-CAT-LacZ* plasmid into HeLa cells. The *pCAG-CAT-LacZ* plasmid carries a *LacZ* (*β-galactosidase*) gene downstream of a chicken beta-actin promoter (*CAG*) and a ‘DNA stuffer’ (*CAT*) flanked by two *loxP* sequences, so that *LacZ* is expressed only when the DNA stuffer is removed by the action of Cre recombinase. Addition of 4-OHT (0.8 µM; +OHT) induced a robust expression of *β*-galactosidase of the co-transfected cells. In contrast, no *β*-galactosidase could be detected in co-transfected cells without 4-OHT treatment (−OHT) (Figure 1). These data indicated that Cre activity was successfully induced in HeLa cells transfected with the *NSE-MerCreMer* and the *pCAG-CAT-LacZ* vectors upon treatment with 4-OHT.

To generate transgenic mice that express Cre recombinase in neurons, *NSE-MerCreMer* was micro-injected into fertilized eggs. A total of 54 mice were born from the micro-injection, and six transgenic founders (4 males and 2 females) were identified by PCR genotyping (Figure 1B). All the six founders transmitted the transgene through germ-line, and 6 transgenic lines were established (Table 1). Brain and intestine contain neurons of the central nervous system (CNS) and the enteric nervous system (ENS), respectively. Therefore, brain and intestine were collected from the F3 transgenic mice (postnatal week-4) of each line, and expression of the transgene was analyzed by RT-PCR and Western blot. Transcript of *MerCreMer* was detected in four transgenic lines by RT-PCR (Figure 1C). Expression of MerCreMer protein in transgenic mice was further confirmed by Western blot using the monoclonal anti-body (anti-ERα), which recognized the ligand binding domain of the estrogen receptor α. Therefore, anti-ERα could recognize both the endogenous ERα and the mutated ERα ligand binding domain in MerCreMer. As shown in Figure 1D, strong bands were detected in uterus and intestine, corresponding to endogenous ERα (67 kDa) and MerCreMer protein (113 kDa), respectively in two transgenic lines (#778, #805). A very weak band (corresponding to 113 kDa) was also observed in brain of mice in transgenic lines (#778, #805) upon prolonged exposure (data not shown). However, MerCreMer protein expression was not detected in the intestine and brain of the other 2 transgenic lines that were found expressing the *MerCreMer* transcript by RT-PCR (date not shown).

**Table 1:** Statistics of the generation of *NSE-MerCreMer* transgenic mice.

Number of mice born	54
Number of founders (% transgenesis)	6 (11.1%) (4♂; 2♀)
Germ-line transmission (% transmission)	6/6 (100%)
Transgene expression^a^	4/6 (RT-PCR); 2/6 (WB)

a , Number of transgenic lines showing expression of transgene in brain and intestine as detected by RT-PCR or Western blot (WB).

**Figure 2:**
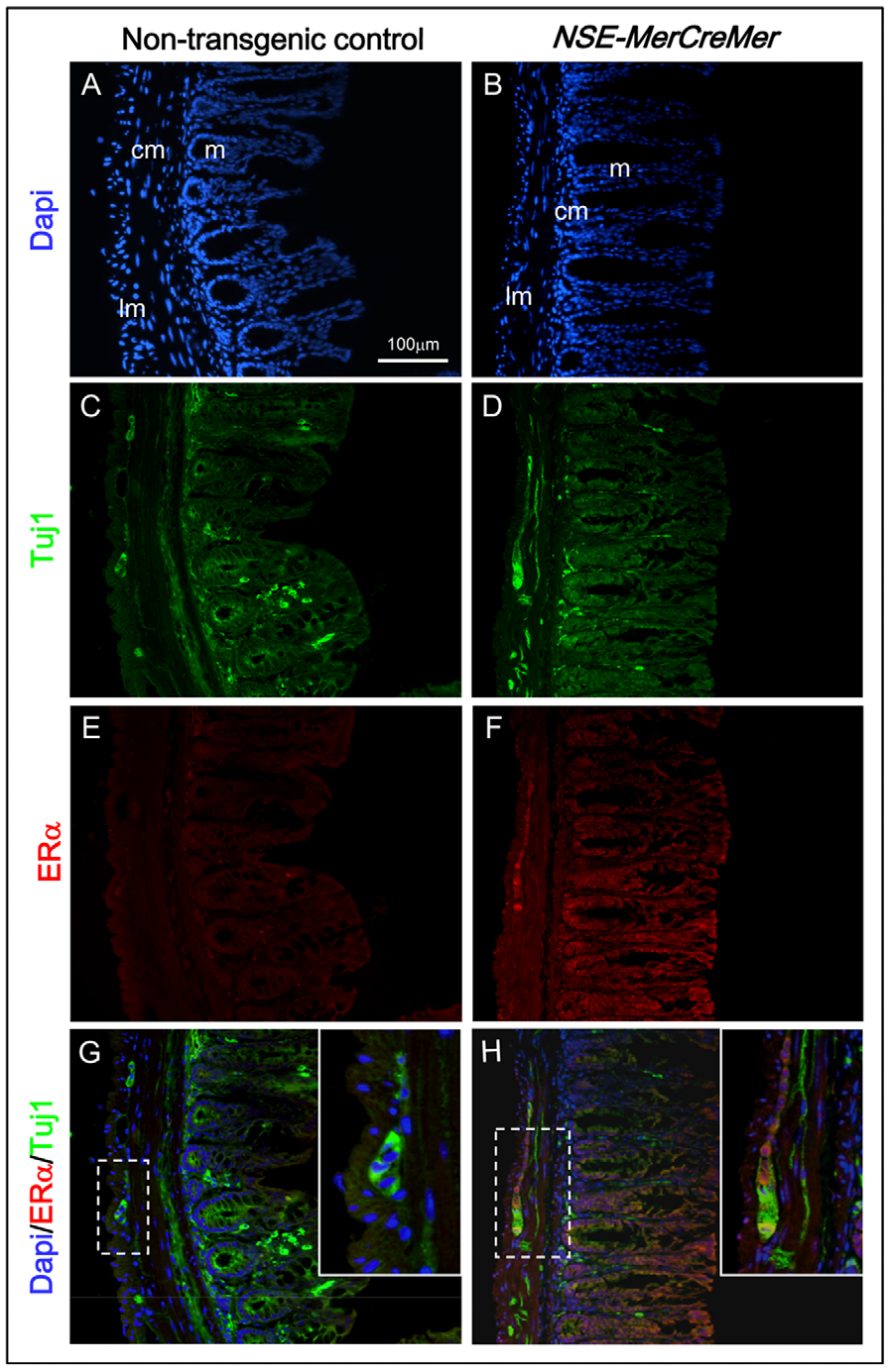
Localization of MerCreMer protein to the enteric neurons in the intestine of *NSE-MerCreMer* transgenic mice. Immuno-reactivity for Tuj1 (green; neuronal marker) was localized to the enteric ganglion plexus at the myenteric region between the circular muscle and the longitudinal muscle of the intestine of non-transgenic mice (C), and transgenic mice (D). In contrast, immuno-reactivity for ERα (red) was only detected at the myenteric plexus in the intestine of transgenic mice (F), but not in non-transgenic mice (E). Superimposed photos of immuno-fluorescence for ERα (red) and Tuj1 (green) of transgenic intestine (H), and non-transgenic intestine (G) showed co-localization of ERα and Tuj1 immuno-reactivity in transgenic intestine. Dotted regions are magnified and showed as insets. Abbreviations: m, mucosa; cm, circular muscle; lm, longitudinal muscle.

Expression of MerCreMer protein in the neurons of the enteric nervous system (ENS) of the intestine in transgenic mice (from lines #778 and #805) was investigated by immuno-fluorescence using anti-bodies against estrogen receptor α (ERα) and Tuj1 (neuron marker). Immuno-reactivity of Tuj1 (green) was localized to the neurons and nerve fiber of the ENS of transgenic and non-transgenic intestines (Figure 2C, D and data not shown). Immuno-reactivity of ERα (red) was detectable at the myenteric ganglion plexus between the circular and the longitudinal muscle layers of the intestine of transgenic mice (Figure 2F). Furthermore, ERα immuno-reactivity was localized to the cytoplasm overlapping with that of neuron marker Tuj1 (Figure 2H) in transgenic intestine, indicating the cytoplasmic expression of MerCreMer in neurons. The absence of ERα immuno-reactivity in non-transgenic control (Figure 2E), further confirmed the specificity of the ERα antibody for the detection of MerCreMer transgenic protein in the transgenic intestine.

### Temporal and Spatial Expression of MerCreMer in NSE-MerCreMer Mice

**Figure 3:**
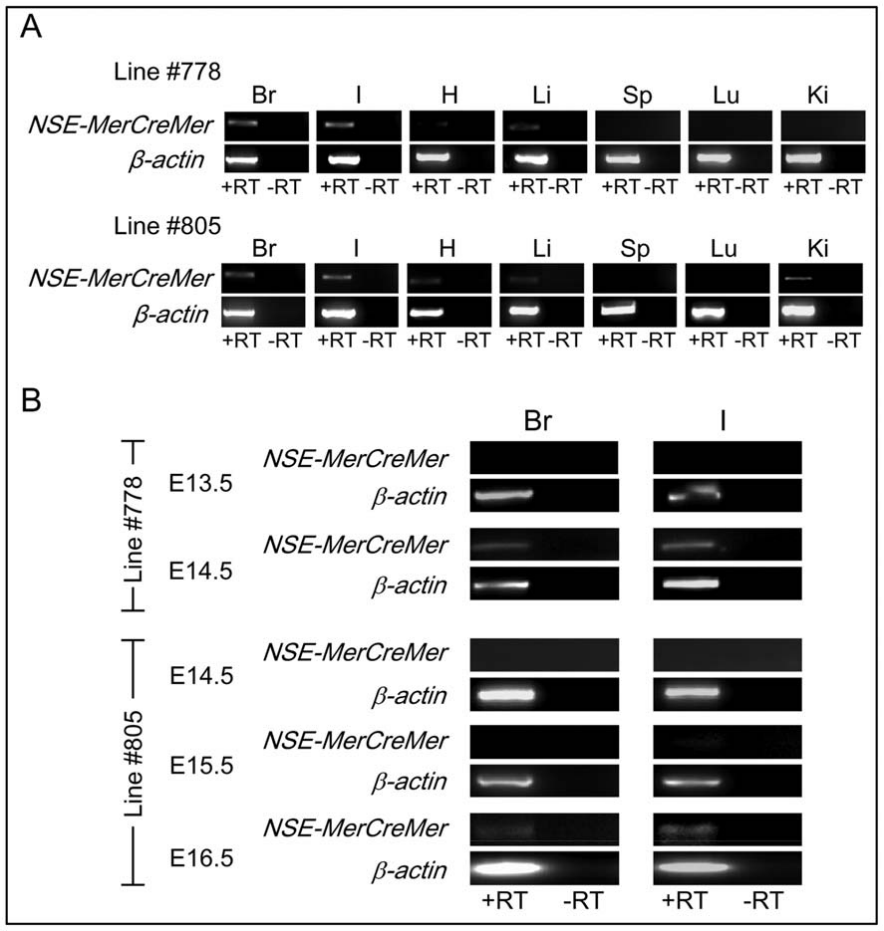
Spatial and temporal expression of transgene in *NSE-MerCreMer* mice. Total RNA was isolated from neural and non-neural tissues from postnatal week-4 transgenic mice (A) or brain and intestine of transgenic embryos of various embryonic stages (B) from lines #778 and #805. Expression of the transgene (*NSE-MerCreMer*) was analyzed by RT-PCR. RT-PCR for mouse *β*-actin (*β*-*actin*) was included to check the integrity of the isolated RNA. Reverse transcriptase was omitted in the first strand cDNA synthesis which served as a negative control (-RT) to ascertain the PCR product was not amplified from genomic DNA. Abbreviations: Br, brain; I, intestine; H, heart; Li, liver; Sp, spleen; Lu, lung; Ki, kidney.

RT-PCR analysis was performed on RNAs isolated from various tissues of postnatal week-4 transgenic mice to investigate if *MerCreMer* transcript was expressed in other tissues besides the central and the enteric nervous systems. High level expression of *MerCreMer* was observed in the brain and intestine of transgenic mice, nevertheless, weak to moderate expressions of *MerCreMer* transgene were detectable in the heart and liver of transgenic mice from line #778; and in the heart, liver and kidney of mice from line #805 (Figure 3A). In line with low level expression of transgene in non-neural tissues as observed in our transgenic mice, the same *NSE* promoter has been previously shown to direct weak level of transgene expression in non-neural tissues [13], [15], [16], [18].

In mouse brain, neurogenesis occurs from E12 to E17 [21], [22]. In mouse intestine, ENS progenitor cells migrate along the gut, proliferate and differentiate into neurons and glia between E10.5 and E14.5 [23]. The expression of *MerCreMer* in brain and intestine of transgenic embryos between E12.5 and E16.5 was evaluated by RT-PCR to determine if the onset of the *NSE-MerCreMer* expression matched with the neurogenesis of the CNS and ENS in embryonic stages. The earliest detectable expression of *MerCreMer* transcript in brain and intestine was at E14.5 for transgenic line #778 (Figure 3B), which correlated with the neurogenesis of CNS and ENS. However, for transgenic line #805, expression of *MerCreMer* in brain and intestine was not detectable till E16.5 (Figure 3B). Transcript of mouse endogenous *NSE* was detectable from E12 onwards [13] and its expression was correlated with synaptogenesis [12], [14]. However, the same *NSE* promoter has been shown to direct transgene expression in the embryonic brain as early as E9.5 [16]. The variations in the embryonic onset of transgene expression among different transgenic lines could be attributable to the (i) influences of the genetic loci at which the transgene integrated in different transgenic lines, and/or (ii) number of copies of the transgene integrated into the genome.

### Tamoxifen Inducible Cre Recombinase Activity in Neurons

**Figure 4:**
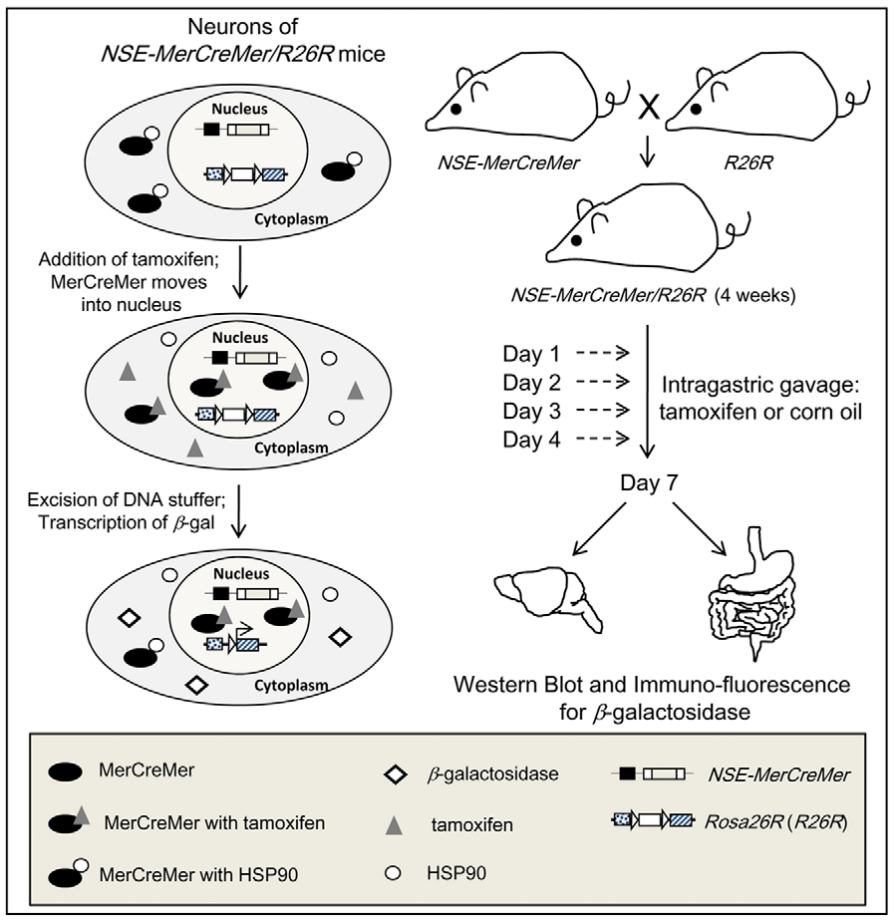
Schematic diagram of transgenic mice crossing and tamoxifen induction of Cre activity. MerCreMer protein in the cytoplasm of the neurons of *NSE-MerCreMer/R26R* mice moves into the nucleus after binding to tamoxifen, mediates the excision of the DNA stuffer. The *LacZ* gene is transcribed. *NSE-MerCreMer* mice were crossed to *Rosa26R* reporter mice (*R26R*) to generate *NSE-MerCreMer/R26R* double transgenic mice. Tamoxifen or corn oil was administered to postnatal week-4 double transgenic mice at day 1, 2, 3 and 4. Brain and intestine were harvested at day 7, and processed for Western blot analysis and immuno-fluorescence staining for *β*-galactosidase.

*NSE-MerCreMer* transgenic mice (from lines #778 and #805) were crossed with *Rosa26R* Cre-dependent *lacZ* reporter mice (*R26R*) (Soriano, 1999) to generate transgenic mice carrying both the *NSE-MerCreMer* and *R26R* transgenic loci (*NSE-MerCreMer/R26R*). Double transgenic mice (postnatal week-4) were divided into two groups (experimental group and control group). For the experimental group, mice were given tamoxifen via intragastric gavage for 4 consecutive days. Corn oil was given to mice in the control group. Mice of both groups were sacrificed 3 days after the last administration, brain and intestine were processed to assay the tamoxifen inducible Cre activity in neurons of *NSE-MerCreMer/R26R* transgenic adults by Western blot analysis and immuno-histochemistry for *β*-galactosidase (Figure 4).

**Figure 5:**
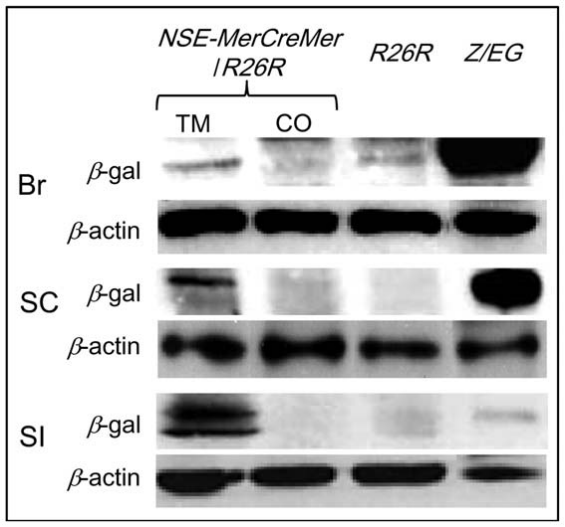
Tamoxifen induction of *β*-galactosidase in the central nervous system and the intestine of *NSE-MerCreMer*/*R26R* mice. Western blot analysis of protein extracted from the brain (Br), spinal cord (SC) and small intestine (SI) of *NSE-MerCreMer*/*R26R* mice treated with tamoxifen (TM) or corn oil (CO); *Rosa26R* mice (*R26R*) and *Z/EG* mice. Except for the small intestine of *Z/EG* mice that 20 µg of protein was loaded, 100 µg of protein was analyzed for all the other samples.

As shown in Figure 5, Western blot analysis with anti-*β*-galactosidase serum demonstrated the presence of *β*-galactosidase in the brain, spinal cord and small intestine of *NSE-MerCreMer/R26R* transgenic mice only after tamoxifen induction, but *β*-galactosidase was not detected in these tissues of the corn oil controls. A weak expression of *β*-galactosidase was detected in the brain of *R26R* mice, which indicated a low level of leaky expression of the *β*-galactosidase from the *Rosa26R* locus.

**Figure 6:**
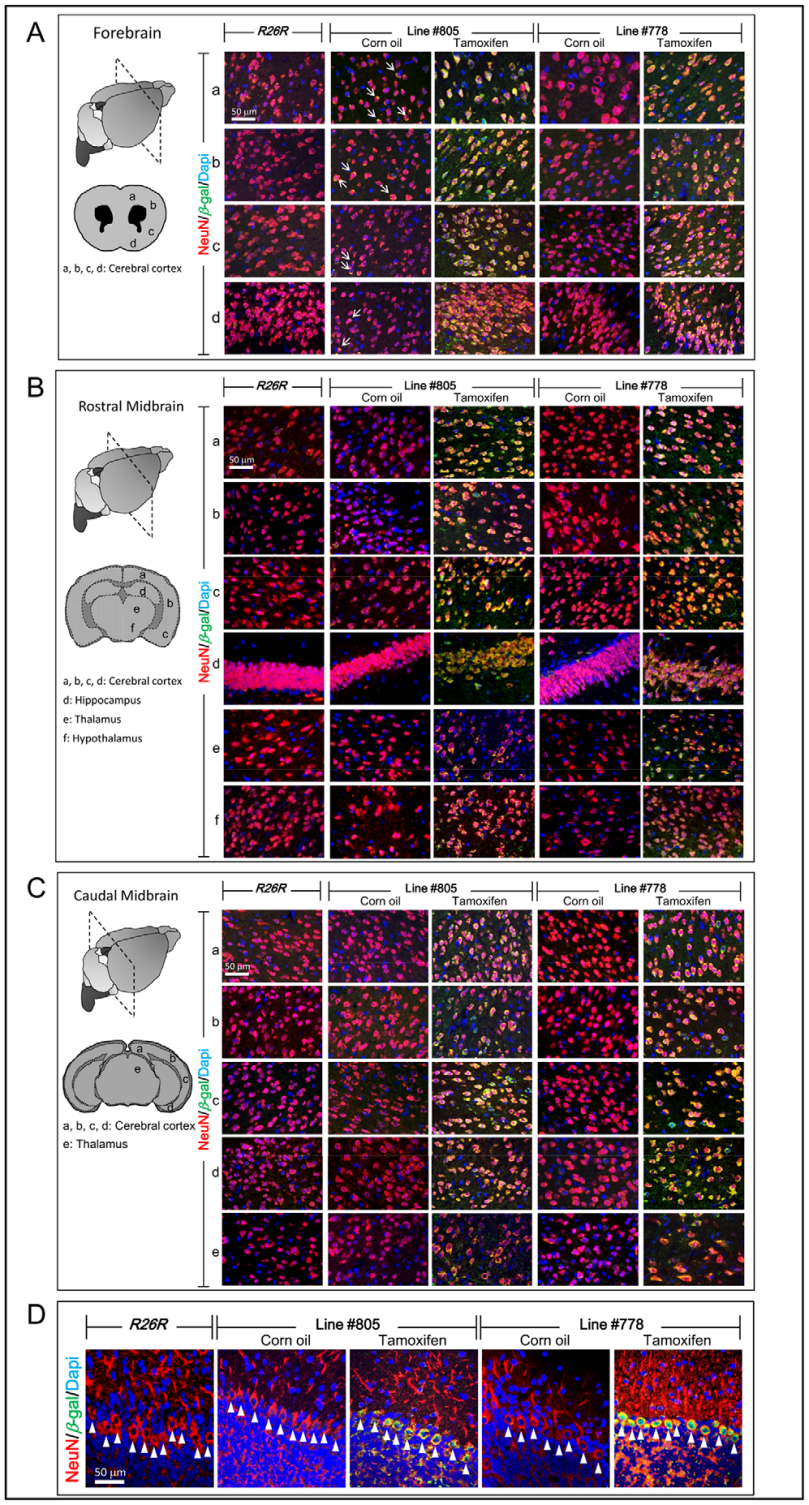
Tamoxifen induction of *β*-galactosidase in neurons of the central nervous system. Coronal sections of the forebrain (A), rostral (B) and caudal (C) midbrain, and cerebellum (D) of *NSE-MerCreMer*/*R26R* double transgenic mice of lines #778 and #805 fed with tamoxifen (TM) or corn oil (CO) and *Rosa26R* (*R26R*) mice were analyzed by co-immunofluorescence staining for *β*-galactosidase (green) and NeuN (red). Superimposed photos of immuno-fluorescence for *β*-galactosidase (green) and NeuN (red) showed co-localization of *β*-galactosidase and NeuN immuno-reactivity in neurons in tamoxifen fed *NSE-MerCreMer*/*R26R* mice. Arrowheads indicated the Purkinje cells in the cortex of the cerebellum (D). Arrows indicated that few neurons in the corn oil treated forebrain cerebral cortex expressed weak *β*-galactosidase (A). Dotted square indicated the plans of the coronal sections being analyzed from different regions of the brain. The respective locations of the superimposed photos being taken from the sections were indicated by letters on the drawing of the sections. At least two *NSE-MerCreMer*/*R26R* mice from each line were analyzed for each treatment groups.

Spatial expression of *β*-galactosidase in the brain of tamoxifen treated *NSE-MerCreMer/R26R* double transgenic mice from both transgenic lines was investigated by co-immuno-fluorescence using anti-sera for *β*-galactosidase and NeuN (neuronal marker). Corn oil treated *NSE-MerCreMer/R26R* mice and *R26R* mice were included as controls to ascertain the specificity of the Cre-mediated induction of *β*-galactosidase upon tamoxifen administration in *NSE-MerCreMer/R26R* mice. Coronal sections from the forebrain, rostral and caudal midbrain, and cerebellum of *NSE-MerCreMer/R26R* mice (both lines #778 and #805; tamoxifen and corn oil treated; at least two mice from each line were analyzed in different treatment groups) and *R26R* mice were co-immuno-stained for *β*-galactosidase (green) and NeuN (red). In tamoxifen treated *NSE-MerCreMer/R26R* mice from both lines, majority of the neurons (NeuN immuno-positive; red) at various regions of the cerebral cortex from the forebrain (Figure 6A), rostral (Figure 6B) and caudal midbrain (Figure 6C) were also immuno-positive for *β*-galactosidase. In addition, neurons at the hippocampus, thalamus and hypothalamus were also immuno-positive for *β*-galactosidase in these tamoxifen treated *NSE-MerCreMer/R26R* mice. In corn oil treated *NSE-MerCreMer/R26R* mouse brains, with the exception that few neurons at the forebrain cerebral cortex of line #805 expressed very low level of *β*-galactosidase (arrows; Figure 6A), neurons at all the other regions of the brain were not immuno-reactive for *β*-galactosidase. No *β*-galactosidase immuno-reactivity was detectable in all the brain sections of *R26R* mice (Figure 6A-C). The weak expression of *β*-galactosidase in the forebrain cerebral cortex of corn oil treated *NSE-MerCreMer/R26R* mice from line #805 was probably attributed to low level of Cre activity in the forebrain region of the mouse line #805. Purkinje cells in the cortex of the cerebellum of the brain from the tamoxifen treated *NSE-MerCreMer/R26R* mice from both lines were also immuno-positive for *β*-galactosidase (arrowheads; Figure 6D). In contrast, Purkinje cells in the cerebellum of corn oil treated *NSE-MerCreMer/R26R* mice and *R26R* mice were not immuno-reactive for *β*-galactosidase.

**Figure 7:**
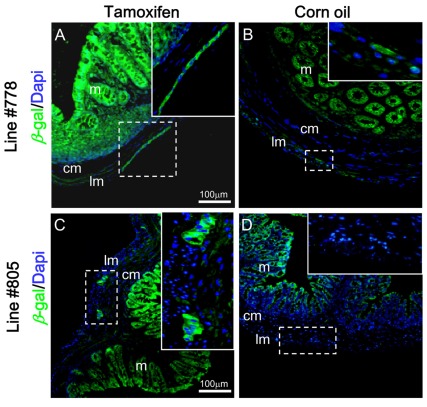
Tamoxifen induction of Cre activity in neurons of the enteric nervous system. Intestine of *NSE-MerCreMer*/*R26R* double transgenic mice of lines #778 and #805 fed with tamoxifen (A, C) or corn oil (B, D) were analyzed by immuno-fluorescence for *β*-galactosidase (green). Dotted regions were magnified and shown as insets. Abbreviations: m, mucosa; cm, circular muscle; lm, longitudinal muscle.

In the intestine, expression of *β*-galactosidase (green) was specifically localized to the myenteric ganglion plexus of tamoxifen treated *NSE-MerCreMer/R26R* double transgenic mice (Figure 7A, C). Very weak or a complete absence of *β*-galactosidase immuno-reactivity was detected at the myenteric ganglion plexus in the corn oil treated double transgenic intestine from transgenic line #778 (Figure 7B), and #805 (Figure 7D).

RT-PCR revealed weak to moderate expression of *MerCreMer* transgene in some of the non-neural tissues from line #778 and line #805 (Figure 3A), immuno-fluorescence staining for *β*-galactosidase were performed on sections prepared from these non-neural tissues of *NSE-MerCreMer/R26R* mice (both lines #778 and #805; tamoxifen and corn oil treated). No specific staining could be detected in these non-neural tissues from either tamoxifen or corn oil treated *NSE-MerCreMer/R26R* mice (data not shown).

In conclusion, we established transgenic mouse lines *NSE-MerCreMer* that express tamoxifen inducible Cre activity, which allow a temporal control of the *Cre*/*loxP* recombination event in neurons. Our transgenic mice will be very useful in studying loss of gene function (Cre-mediated gene deletion) and/or gain of gene function (Cre-mediated gene expression) by Cre-mediated recombination in neurons, which results in embryonic lethality and precludes investigation of gene functions in neurons through later stages of development and in adult. Furthermore, as a gene expressed in neurons at different developmental stages may play distinct roles, *NSE-MerCreMer* mice allow investigation of different functions of a gene in neurons at different stages of nervous system development, providing critical information on the pathogenesis of neurodegeneration in normal aging and pathological conditions, which has been obsessing many individuals and levying a heavy burden to society.

## Materials and Methods

### Generation of Transgenic Construct NSE-MerCreMer

The 1.8 kb promoter of the rat neural specific enolase (NSE) gene was subcloned from the *NSE-Cre* transgenic construct [16] 5′ upstream of the cDNA encoding the MerCreMer protein [9]. The insert of the transgenic construct *NSE-MerCreMer* was completely sequenced to ascertain that no mutation was introduced during the cloning steps.

### Cell Culture and Transfection

HeLa cells were cultured in DMEM containing 10% fetal calf serum (Clontech, CA, USA), 100 U/ml penicillin, 100 µg/ml streptomycin, 2 mM L-glutamine, and 1 mM sodium pyruvate in 5% CO_2_ at 37°C. HeLa cells (3x10^5^) were plated in each well of a 24-well tissue culture plate 24 hours before co-transfection with *NSE-MerCreMer* construct and *pCAG-CAT-LacZ* plasmid (0.5 µg each) using Lipofectamine 2000 (Invitrogen™) according to the manufacturer’s protocol. Cre activity was induced by the addition of 4-hydroxy-tamoxifen (4-OHT; 0.8 µM as final concentration) to the culture and incubation for 27 hours. Cells were then fixed in 4% paraformaldehyde/PBS (PFA/PBS; 10 minutes, room temperature), washed with PBS and stained for *β*-galactosidase activity in X-gal staining solution containing 5 mM K_3_Fe(CN)_6_, 5 mM K_4_(CN)_6_, 2 mM MgCl_2_, 0.01% Sodium deoxycholate, 0.02% NP-40, 1 mg/ml X-gal in PBS at 37°C for 3 hours.

### Generation of Transgenic Line

The DNA insert of *NSE-MerCreMer* construct was released by *NotI* digestion, purified and micro-injected into fertilized oocytes collected from super-ovulated (*CBA/129*) *F1* hybrid mouse. The micro-injected fertilized eggs were transferred to pseudo-pregnant wild type *ICR* foster mothers. *NSE-MerCreMer* transgenic founder mice were backcrossed to (*CBA/129*) *F1* hybrid mice for germ-line transmission to establish transgenic lines. All experimental procedures were approved by the Committee on the Use of Live Animals at the University of Hong Kong (approval CULTRA 1044-05).

### Genotyping of Transgenic Mice

Genomic DNA was extracted from 2 mm tail clip using PBND extraction method (http://www.jax.org/imr/tail_nonorg.html). In brief, mouse-tail was digested with 40 µg Proteinase K (Invitrogen™) in 200 µl PBND buffer at 55°C for 16 hours. After heating at 96°C for 10 minutes to inactivate the Proteinase K, the tail digest was used as template DNA for PCR analysis. For the detection of the *MerCreMer* transgene, the following primers were used: *CreF1* (5′-CGT ACT GAC GGT GGG AGA AT-3′) and *CreR1* (5′-TGC ATG ATC TCC GGT ATT GA-3′). PCR reaction was performed in PCR buffer (25 µl) containing 0.2 mM dNTP (Promega), forward and reverse primer (0.2 µM each), template DNA (1 µl), DMSO (5%; v/v, Merck), MgCl_2_ (4 mM) and 0.25 µl of Ampli Taq Gold™ (Roche). Amplication was performed as follows: initial denaturation: 94^o^C for 7 minutes, 40 PCR cycles: 94°C; 30 seconds, 54°C; 30 seconds, 72°C; 30 seconds, final extension: 72^o^C; 10 minutes. Amplification of *NSE-MerCreMer* transgene generated a 374 bp DNA fragment.

### Western Blot Analysis

Brain and small intestine were harvested from postnatal week-4 *NSE-MerCreMer* and non-transgenic mice, homogenized in 1 ml of lysis buffer (Cell signaling) containing protease inhibitor (20 µl; Roche), 1 mM DTT (USB) and 0.1 mM PMSF (USB) on ice. The supernatant was collected by centrifugation (13,000 rpm, 15 minutes, 4^o^C). Protein (30 µg) of each samples were separated by electrophoresis in 8% (w/v) polyacrylamide gel, and were electro-transferred onto PVDF membrane (Millipore). After blocking in TBS-T (50 mM Tris-HCl, pH 7.6, 150 mM NaCl, 0.1% Tween 20) with 10% non-fat milk (w/v) for 16 hours at 4°C, the membrane was incubated with anti-ERα serum (1∶100 dilution in blocking solution; Ab-10; LabVision) for 16 hours at 4°C. After TBS-T rinse (3×15 minutes), and an incubation in HRP-conjugated secondary antibody (1∶5000 dilution; P0399; Dako; 1 hour, room temperature), the membrane was washed in TBS-T. Signal was visualized by chemiluminescence (GE Health Amersham ECL Plus Western Blotting Detection System) and exposure to X-ray film (Kodak). For reprobing, membrane was stripped by heating at 60°C for 30 minutes in 10 mM Tris-HCl pH 6.8; 2% SDS (w/v); 100 mM 2-mercaptoethanol, followed by an incubation with anti-*β*-actin serum (1∶5000; AC-74; Sigma) at room temperature for 1 hour. After TBS-T rinse, signal was visualized by ECL.

To detect the expression of *β*-galactosidase in the brain, spinal cord and small intestine of *NSE-MerCreMer* mice upon tamoxifen induction, protein of each samples from week-4 *NSE-MerCreMer* mice (tamoxifen or corn oil treated; at least two mice were analyzed from each line in each treatment groups) and *Rosa26R* mice were prepared as described above. Samples from the week-4 *Z/EG* mice (Tg(ACTB-Bgeo/GFP)21Lbe/J; The Jackson Laboratory) that expressed *β*-galactosidase constitutively were included as a positive control. Protein (100 µg) of each samples were separated by electrophoresis in 6% (w/v) polyacrylamide gel, and were electro-transferred onto PVDF membrane. After blocking in TBS-T with 5% non-fat milk (w/v) for 2.5 hours at room temperature, the membrane was incubated with anti-*β*-galactosidase serum (1∶5000 dilution; ab616; Abcam) for 16 hours at 4°C. After TBS-T rinse, and an incubation in HRP-conjugated secondary antibody (1∶5000 dilution; P0399; Dako; 1 hour, room temperature), the membrane was washed in TBS-T. Signal was visualized by chemiluminescence (GE Health Amersham ECL Plus Western Blotting Detection System) and exposure to X-ray film (Kodak). Membrane was stripped and reprobed with anti-*β*-actin serum as described above.

### RT-PCR Analysis

Total RNA was isolated using TRIzol (Invitogen™) according to manufacturer’s protocol. RNA (1 µg) was treated with RQ1 RNase-Free Dnase (Promega) to remove genomic DNA. First-strand cDNA was synthesized according to the protocol provided by the Reverse Transcription System (Promega). Transcript of *MerCreMer* was detected by PCR using *CreR1* and *CreF1* primers pair. PCR amplification for mouse *β-actin* using *β-actin* specific primers: *β*-*actinF1* (5′-GAG AGG GAA ATC GTG CGT GAC-3′) and *β*-*actinR1* (5′-AGC TCA GTA ACA GTC CGC CTA-3′) was included as internal control to test the integrity of RNA. PCR amplification was performed as follow: 94^o^C for 7 minutes, 40 cycles of (94°C for 45 seconds, 55°C for 45 seconds, 72°C for 45 seconds), 72^o^C for 10 minutes. Amplification of *β*-*actin* generated a 534 bp fragment.

### Immuno-fluorescence

Brain and intestine were fixed in 4% PFA in PBS at 4^o^C for overnight. After PBS (Phosphate buffered saline pH 7.4) rinse, the specimens were incubated in 10% sucrose solution for 1 hour at 4^o^C and then 30% sucrose solution at 4^o^C for 24 hours. The specimens were embedded and frozen in O.C.T. (Tissue-Tek). Transverse sections (6 µm in thickness) were prepared and mounted onto microscopic glass slides coated with TESPA (3-aminopropyl-triethoxysilane; Sigma). Antigen was retrieved by incubating in 10 mM sodium citrate buffer (pH 6.0) at 95°C for 10 minutes. After blocking in PBS-T (PBS with 0.1% Triton) supplemented with 10% normal goat serum (Dako) for 1 hour at room temperature, sections were incubated with either anti-*β*-galactosidase (1∶200; ab8361; Abcam) plus anti-NeuN (1∶100; MAB377; Chemicon) or anti-ERα (1∶100; Ab-10; LabVision) plus anti-Tuj1 (1∶500; MMS-435P; Covance) in PBS-T for overnight at 4^o^C. The sections were washed in PBS-T and incubated with appropriate fluorochrome conjugated secondary antibody (1∶200; Invitrogen™) at room temperature for 2 hours. After PBS-T wash, sections were mounted in DAPI containing anti-fade solution (Vector Laboratories., Inc.). Images were taken with Nikon Eclipse 80i microscope mounted with SPOT RT3 microscope digital camera (DIAGNOSTIC instruments, Inc.), and photos were compiled using Adobe Photoshop 7.

### Tamoxifen Induction

Tamoxifen stock (20 mg/ml of corn oil) was prepared by warming tamoxifen (Sigma) in corn oil at 65^o^C (protected from light) until completely dissolved. Tamoxifen (0.15 mg per gram of body weight for four consecutive days) was administered by intragastric gavage to adult mice. For control, corn oil was given instead of tamoxifen.
